# Utility of Cardiac Magnetic Resonance to assess association between admission hyperglycemia and myocardial damage in patients with reperfused ST-Segment Elevation Myocardial Infarction

**DOI:** 10.1186/1532-429X-10-2

**Published:** 2008-01-15

**Authors:** Alexandre Cochet, Marianne Zeller, Alain Lalande, Isabelle L'Huillier, Paul M Walker, Claude Touzery, Bruno Verges, Jean-Eric Wolf, François Brunotte, Yves Cottin

**Affiliations:** 1 Unité d'IRM, CHU Hôpital d'enfants, Boulevard du Maréchal de Lattre de Tassigny, 21034 Dijon, France; 2 Institut Fédératif de Recherche Santé STIC, Université de Bourgogne, France; 3 Département de Cardiologie, CHU Bocage, Boulevard du Maréchal de Lattre de Tassigny, 21034 Dijon, France; 4 Département d'Endocrinologie, CHU Bocage, Boulevard du Maréchal de Lattre de Tassigny, 21034 Dijon, France

## Abstract

**Aims:**

to investigate the association between admission hyperglycemia and myocardial damage in patients with ST-segment elevation myocardial infarction (STEMI) using Cardiac Magnetic Resonance (CMR).

**Methods:**

We analyzed 113 patients with STEMI treated with successful primary percutaneous coronary intervention. Admission hyperglycemia was defined as a glucose level ≥ 7.8 mmol/l. Contrast-enhanced CMR was performed between 3 and 7 days after reperfusion to evaluate left ventricular function and perfusion data after injection of gadolinium-DTPA. First-pass images (FP), providing assessment of microvascular obstruction and Late Gadolinium Enhanced images (DE), reflecting the extent of infarction, were investigated and the extent of transmural tissue damage was determined by visual scores.

**Results:**

Patients with a supramedian FP and DE scores more frequently had left anterior descending culprit artery (p = 0.02 and <0.001), multivessel disease (p = 0.02 for both) and hyperglycemia (p < 0.001). Moreover, they were characterized by higher levels of HbA_1c _(p = 0.01 and 0.04), peak plasma Creatine Kinase (p < 0.001), left ventricular end-systolic volume (p = 0.005 and <0.001), and lower left ventricular ejection fraction (p = 0.001 and <0.001).

In a multivariate model, admission hyperglycemia remains independently associated with increased FP and DE scores.

**Conclusion:**

Our results show the existence of a strong relationship between glucose metabolism impairment and myocardial damage in patients with STEMI. Further studies are needed to show if aggressive glucose control improves myocardial perfusion, which could be assessed using CMR.

## Introduction

Admission hyperglycemia is associated with increased short and long term risk of death in patients with ST-segment elevation myocardial infarction (STEMI), independently of the presence of Diabetes Mellitus [1-3]; this increased mortality might be explained by a higher incidence of congestive heart failure and cardiogenic shock [2,4]. Moreover, a recent study suggested an association between admission hyperglycemia and the no-reflow phenomenon as assessed by myocardial contrast echocardiography [5]. In contrast, the association between hyperglycemia and the extent of myocardial infarct size is less clear [6-8].

Cardiac Magnetic Resonance (CMR) with a gadolinium-based contrast agent is considered to accurately assess myocardial perfusion and function abnormalities after STEMI. First pass (FP) perfusion provides a valuable assessment of the extent of microvascular obstruction (MO) [9], and Late Gadolinium Enhancement (DE) reflects the extent of myocardial infarction [10,11]. However, no data are available on the relationship between admission glycemia and CMR data in the setting of STEMI.

The objective of the present study was to investigate the relationship between blood glucose levels taken on admission and myocardial perfusion parameters assessed by contrast-enhanced CMR in patients after reperfusion of an acute STEMI.

## Methods

### Study population

One hundred and thirty one consecutive patients presenting with a first acute STEMI and who underwent primary percutaneous coronary intervention (PCI, angiography and/or stenting) for arteries exhibiting Thrombolysis In Myocardial Infarction (TIMI) flow grade < 3, within 24 hours after symptom onset, were included in the study. Patients with TIMI flow <3 on the culprit lesion after PCI, or who were hemodynamically unstable or with a contraindication for CMR were excluded from the study. The diagnosis of STEMI was based on prolonged chest pain, ST-segment elevation ≥ 2 mm in at least two contiguous electrocardiographic (ECG) leads, and a more than three fold increase in serum creatine kinase (CK) levels [12,13].

Fourteen patients with a TIMI flow <3 post-PCI and 4 patients with contraindications for CMR were excluded from the study

Therefore, the study population consisted of 113 patients (91 men and 22 women, mean age 57 ± 14 years).

All participants gave written consent before entering the study, in accordance with a protocol approved by the institutional ethics committee.

### Data Collection

Demographic data, age, gender, Body Mass Index (weight/height^2^, kg/m^2^), clinical history of hypertension, diabetes mellitus, hypercholesterolemia, smoking, previous PCI and/or Coronary Artery Bypass Graft and peripheral arterial disease were recorded as were on-admission clinical data (Killip class, ECG and hemodynamic parameters). Heart failure was defined by Killip class > 1. Location of MI was determined according to ECG, coronary angiographic and CMR data; no discordance was observed between the 3 methods. Current treatment before hospitalization was also recorded. Blood samples were taken at admission to measure glucose and C-Reactive Protein levels. HbA_1c_, HDL-Cholesterol, LDL-Cholesterol and triglyceride levels were assessed from fasting blood sample taken on the first morning after admission. Peak plasma levels of Creatine Kinase (CK) were also evaluated and dichotomized at ten times the upper normal limit for more clinical relevance. Baseline serum creatinine clearance was also evaluated by the Cockcroft-Gault formula [14].

### CMR protocol

Patients were examined 3 to 7 days after STEMI. Contrast-enhanced CMR was performed on a 1.5 T whole-body MR imager (Magnetom Vision, Siemens GmbH, Erlangen, Germany), using a phased array body coil. Left ventricular (LV) long-axis scout images were obtained to determine the orientation of the LV short axis imaging planes.

For LV function evaluation, cine-CMR was performed using a breath-hold ECG-gated gradient echo sequence (FLASH 2D), with the following acquisition parameters: repetition time (TR) 9 ms, echo time (TE) 4.4 ms, pulse flip angle (α) 15°, 9 lines per segment, field of view (FOV) of 350 to 400 mm. The acquisition matrix varied from 108 × 256, (12 cardiac cycles), to 144 × 256, (16 cardiac cycles), depending on the ability of patient to hold his breath. A series of short-axis slices (thickness of 5 mm with a gap between slices of 10 mm) was defined from the base of the heart to the apex [15]. The image of the heart was taken into account at end-systole and end-diastole times. A temporal resolution of 50 ms was obtained with the "view-sharing" technique [16].

**Figure 1:**
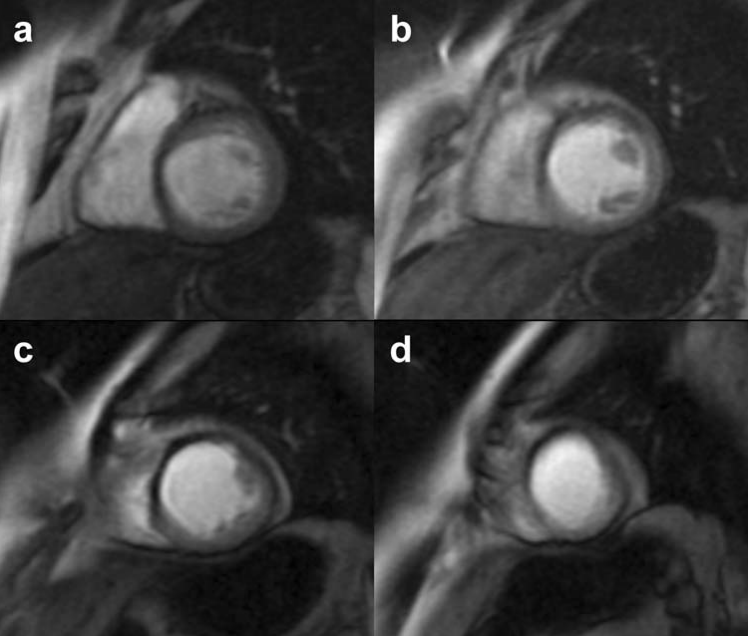
Short-axis gradient-echo CMR images at basal (**a**), mid-ventricular (**b, c**) and distal (**d**) levels, during first pass of a bolus of gadolinium-DTPA, showing a localized microvascular obstruction in the antero-septal territory.

For LV perfusion evaluation, a first-pass ECG-gated gradient echo sequence was performed (T1-weighted turboFLASH sequence) after injection of a bolus of gadolinium-diethylenetriamine pentaacetic acid (Magnevist, Schering-AG, Berlin, Germany) into a brachial vein at 0.1 mmol/Kg; the acquisition parameters were as follows: inversion time (TI) 400 ms, TR 3.5 ms, TE 1.7 ms, FOV 350 to 400 mm, and acquisition matrix 96 × 128, interpolated to 256 × 256. A time delay buffer at the end of the sequence was adjusted in order to acquire one image every two cardiac cycles. Three to 5 short-axis slices were obtained from the base of the heart to the apex, with the same plane as for the functional study (thickness of 12 mm with a gap between slices of 3 mm) (Figure 1) [17].

**Figure 2:**
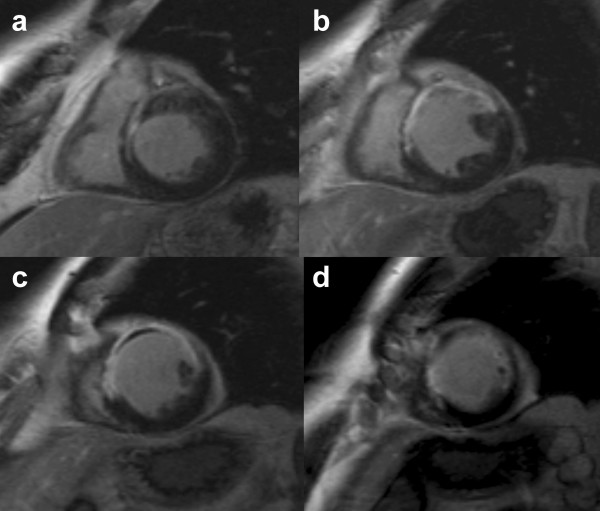
Short axis basal (**a**), mid-ventricular (**b, c**) and distal (**d**) segmented Inversion Recovery gradient echo MR images 10 minutes after bolus injection of Gadolinium-DTPA, showing a large region of myocardial damage (hyperenhancement surrounding region of persistent hypoenhancement) in the antero-septal territory (same patient than Figure 1).

Ten minutes after bolus injection, delayed images were acquired using a segmented T1-weighted inversion-recovery turboFLASH sequence [18], with the following parameters: TR = 2 cardiac cycles; TE = 3.4 ms; α = 25°; FOV = 350 mm; acquisition matrix = 165 × 256, interpolated to 256 × 256, with a variable TI adjusted for each patient studied in order to optimize the nulling of normal myocardium [19]. This time delay was usually about 250 ms (Figure 2). The image planes were the same as for functional and first-pass studies (thickness of 8 mm with a gap between slices of 7 mm).

### CMR data analysis

A 17-segment model was used for the data analysis of perfusion studies [20]. Left ventricular end-diastolic volume (EDV) and end-systolic volume (ESV) were calculated from short-axis views using a method previously described [15]. Left ventricular ejection fraction (LVEF) was calculated with the following formula: LVEF = (EDV-ESV)/EDV.

For each patient, perfusion data were analysed and visual scores were independently attributed by two expert investigators blinded to clinical data. These scores were given according to the signal intensity observed on early images (First-pass images: FP) for 16 segments (the apical segment was not evaluated on early images, because no long axis slice was obtained), and late images (delayed enhanced images: DE, 10 minutes after injection) for each of the 17 segments (vertical and horizontal long-axis slices made it possible to evaluate the apical segment). Early regional hypoenhancement and late regional hyperenhancement were scored with a scheme based on the transmural extent of contrast enhanced tissue within each segment (0 = no hypo or hyperenhancement; 1 = 1–25% hypo or hyperenhancement; 2 = 26–50% hypo or hyperenhancement; 3 = 51–75 hypo or hyperenhancement; 4 = 76–100% hypo or hyperenhancement). The two scores, FP, which reflects microvascular obstruction and DE, which reflects myocardial infarct size, were determined by calculating the sum of the scores of all of the segments (16 for FP, 17 for DE). Thus, the scores ranged from 0 to 64 for FP, and from 0 to 68 for DE images [21]. Some areas, known as no-reflow zones, have major micro-vascular damage and are characterized by a persistent hypoenhanced area even on delayed images [22]. Thus, the observers were requested to include the no-reflow zones in the extent of infarction (DE score).

Concordance between expert investigators was satisfactory (interobserver variability <5%). In cases of disagreement, the final decision was taken by consensus.

### Statistical analysis

Continuous data were expressed as median (25^th ^percentile – 75^th ^percentile) and dichotomous data as numbers (percentages).

Patients were categorized according to the median values of FP and DE scores. Comparison between groups was made using the Student t test or the Mann-Whitney test for continuous variables and the Chi square test for discrete variables. Admission glycemia was dichotomized according to presence of hyperglycemia. Hyperglycemia was defined as glucose levels on admission of ≥ 7.8 mmol/l (140 mg/dl), in accordance with the guidelines of the American Diabetes Association [23]. Patients were also categorized according to the presence of hyperglycemia at admission, with regard to CMR data.

Multivariate logistic regression analysis was performed to test for independent predictors of high perfusion scores (> median value). The following variables, with a p value of less than 0.1 on univariate analysis were included in the models: left anterior descending culprit artery, multivessel disease, TIMI 0 or 1 before PCI, hyperglycemia, HbA_1c_, peak CK level, ESV and LVEF. Moreover, age and sex were also included in the models.

The Spearman rank test was performed to analyse the relationship between glycemia and perfusion scores.

All the tests were two-sided and a p value <0.05 was considered as significant.

Statistical analysis was performed with Sigmastat version 2.3 (Statistical Software, SPSS Inc, USA).

## Results

### Determinants of microvascular obstruction (FP score)

Characteristics of the patients according to the median value of the FP score are shown in Tables 1 and 2. Patients with high FP score (>median) more frequently had left anterior descending culprit artery (p = 0.02), multivessel disease (p = 0.02) and hyperglycemia (p < 0.001) than patients with low FP score. Moreover, the level of HbA_1c _(p = 0.01), peak CK (p < 0.001), ESV (p = 0.005) and DE score (p < 0.001) were higher in patients with a high FP score. In contrast, LVEF was lower in the group with a high FP score (p < 0.001).

**Table 1:** Risk factors, biological data and medications according to FP and DE scores: n (%) or median (25^th ^and 75^th ^percentile)

	**FP score ≤ median (n = 57)**	**FP score > median (n = 56)**	**p**	**DE score ≤ median (n = 57)**	**DE score > median (n = 56)**	**p**
**Risk factors**						
Age (years)	56 (48–69)	56 (49–68)	0.66	56 (51–68)	57 (48–68)	0.98
Male	43 (75)	48 (86)	0.25	44 (77)	47 (84)	0.51
BMI (kg/m^2^)	25 (24–28)	26 (24–28)	0.35	25 (24–29)	25 (24–28)	0.54
Hypertension	25 (44)	16 (29)	0.14	22 (39)	19 (34)	0.75
Diabetes	5 (9)	7 (12)	0.74	6 (11)	6 (11)	0.79
Hypercholesterolemia	19 (33)	23 (41)	0.51	23 (40)	19 (34)	0.61
Smoking	23 (40)	29 (52)	0.30	22 (39)	30 (54)	0.42
Previous PCI or CABG	2 (4)	2 (4)	1.00	3 (5)	1 (2)	0.62
Peripheral arterial disease	2 (4)	2 (4)	1.00	2 (4)	2 (4)	1.00
						
**Biological data**						
Hyperglycemia (≥ 7.8 mmol/l)	17 (30)	35 (63)	<0.001	16 (28)	36 (64)	<0.001
Glycemia (mmol/l)	7.0 (6.1–8.4)	8.4 (6.9–9.7)	0.001	6.9 (5.9–8.2)	8.4 (7.0–9.6)	<0.001
HbA_1c _(%)	5.4 (5.2–5.7)	5.7 (5.4–6.3)	0.01	5.5 (5.2–5.7)	5.7 (5.4–6.3)	0.04
Peak CK >10 fold UNL	21 (37)	45 (80)	<0.001	17 (30)	49 (88)	<0.001
CRP (mg/l)	2.2 (1.7–10.0)	4.6 (2.0–11.0)	0.48	2.5 (2.0–11.0)	4.7 (2.0–9.0)	0.81
Creatinine clearance (ml/mn)	87 (73–116)	92 (75–111)	0.48	92 (74–117)	88 (72–111)	0.77
HDL-Cholesterol (mmol/l)	1.04 (0.91–1.33)	1.17 (0.96–1.41)	0.15	1.08 (0.91–1.33)	1.17 (0.94–1.34)	0.50
LDL-Cholesterol (mmol/l)	2.99 (2.49–3.73)	3.01 (2.49–3.90)	0.74	2.93 (2.47–3.79)	3.19 (2.56–3.84)	0.54
Triglyceride (mmol/l)	1.42 (0.92–2.5)	1.29 (0.97–1.73)	0.22	1.45 (1.01–2.4)	1.26 (0.90–1.79)	0.08
						
**Current treatment**						
Statin	6 (11)	8 (14)	0.75	6 (11)	8 (14)	0.75
Insulin	1 (2)	0 (0)	0.99	1 (2)	0 (0)	0.99
ACE inhibitor	6 (11)	3 (5)	0.51	3 (5)	6 (11)	0.47
Beta Blocker	10 (18)	6 (11)	0.44	9 (16)	7 (13)	0.82
Oral antidiabetic	2 (4)	4 (7)	0.66	2 (4)	4 (7)	0.66
						
**Acute medications**						
Gp IIb/IIIa receptor blocker	30 (53)	23 (41)	0.30	24 (42)	29 (52)	0.40
Antiplatelet	48 (84)	52 (93)	0.25	48 (84)	52 (93)	0.25

ACE = Angiotensin-Converting Enzyme; BMI = Body Mass Index; CABG = Coronary Artery Bypass Graft; CK = Creatine Kinase; CRP = C-Reactive Protein; PCI = Percutaneous Coronary Intervention; SBP = Systolic Blood Pressure; UNL = Upper Normal Limit.

**Table 2:** Clinical data, coronary angiographic data, and CMR data according to FP and DE scores: n (%) or median (25^th ^and 75^th ^percentile)

	**FP score ≤ median (n = 57)**	**FP score > median (n = 56)**	**p**	**DE score ≤ median (n = 57)**	**DE score > median (n = 56)**	**p**
**Clinical data**						
Heart failure	8 (14)	3 (5)	0.22	6 (11)	5 (9)	0.98
Heart rate (beats/min)	75 (65–84)	75 (61–80)	0.49	76 (64–84)	75 (62–80)	0.39
Blood pressure						
SBP (mm/Hg)	140 (120–155)	140 (120–160)	0.84	140 (120–153)	140 (120–160)	0.87
DBP (mm/Hg)	84 (70–95)	80 (70–99)	0.85	84 (70–94)	80 (70–100)	0.90
Heart rate-SBP product	10477 (8100–12775)	9660 (7874–12600)	0.29	10077 (7800–12915)	9975 (8000–12464)	0.29
						
**Coronary angiographic data**						
Culprit artery						
Left anterior descending	20 (35)	33 (59)	0.02	16 (28)	37 (66)	<0.001
Left circumflex	7 (12)	5 (9)	0.79	9 (16)	3 (5)	0.41
Right coronary	30 (53)	18 (32)	0.04	32 (56)	16 (29)	0.006
Location of lesion (% proximal/mid/distal)	30/58/12	36/55/9	0.79	26/64/10	40/49/11	0.28
TIMI 0/1 pre-PCI in culprit artery	35 (61)	42 (75)	0.18	31 (54)	46 (82)	0.003
Multivessel disease	19 (33)	30 (54)	0.02	19 (33)	30 (54)	0.02
Stenting	56 (98)	55 (98)	0.48	55 (96)	56 (100)	0.48
Stent length > 15 mm	17 (30)	19 (34)	0.79	18 (32)	18 (32)	0.89
Stent width > 3 mm	15 (26)	13 (23)	0.87	15 (26)	13 (23)	0.87
IABP use	1 (2)	2 (4)	0.99	0 (0)	3 (5)	0.24
Time to reperfusion (min)	205 (145–399)	187 (130–265)	0.19	189 (134–339)	190 (130–265)	0.43
						
**CMR data**						
FP score	2 (0–4)	13 (9–19)		2 (0–6)	11 (7–15)	<0.001
DE score	10 (4–15)	20 (16–28)	<0.001	9 (4–12)	23 (18–29)	
LVEF (%)	54 (48–59)	46 (38–56)	0.001	55 (48–60)	44 (37–55)	<0.001
EDV (ml)	132 (117–165)	154 (130–177)	0.06	132 (117–160)	155 (130–178)	0.01
ESV (ml)	66 (50–77)	77 (59–100)	0.005	57 (48–74)	83 (63–103)	<0.001

DBP = Diastolic Blood Pressure; DE = Delayed-Enhancement; EDV = End Diastolic Volume; ESV = End Systolic Volume; FP = First Pass; IABP = Intra-Aortic Balloon Pumping; LVEF = Left Ventricular Ejection Fraction; PCI = Percutaneous Coronary Intervention; SBP = Systolic Blood Pressure.

Multivariate analysis showed that peak CK (odds ratio [OR] 4.2; 95% confidence interval [CI] 1.6–11.1; p = 0.004) and hyperglycemia (OR 3.8; 95% CI 1.4–9.9; p = 0.007) were independent predictors of high (supra-median) FP scores. However, when DE score was included in the multivariate model, only DE remained an independent predictor of high FP score (Table 3).

**Table 3:** CMR data according to admission hyperglycemia. All values are expressed as median (25^th ^– 75^th ^percentile)

	**Without hyperglycemia (n = 61)**	**With hyperglycemia (n = 52)**	**p**
FP score	4 (0–8)	10 (5–16)	<0.001
DE score	11 (6–19)	18 (14–26)	<0.001
LVEF (%)	53 (43–59)	50 (43–56)	0.38
EDV (ml)	148 (125–170)	142 (117–173)	0.23
ESV (ml)	69 (55–84)	68 (51–95)	0.85

DE = Delayed-Enhancement; EDV = End Diastolic Volume; ESV = End Systolic Volume; FP = First Pass; LVEF = Left Ventricular Ejection Fraction.

### Determinants of myocardial infarct size (DE score)

Patients with high DE scores (> median) had increased risk of left anterior descending culprit artery (p < 0.001), TIMI 0 or 1 before PCI in culprit artery (p = 0.003) and hyperglycemia (p = <0.001) (Tables 1 and 2). HbA_1c _(p = 0.04), peak CK (p < 0.001), EDV (p = 0.01), ESV (p < 0.001) and FP score (p < 0.001) were also increased in this group. In contrast, LVEF (p < 0.001) was significantly decreased in patients with higher DE scores. Multivariate analysis showed that hyperglycemia (OR 10.4; 95% CI 2.8–40.3; p < 0.001), left anterior descending culprit artery (OR 6.6; 95% CI 1.7–25.4; p = 0.006) and peak CK (OR 19.2; 95% CI 4.8–76.7; p < 0.001) were independent predictors of high DE scores. When FP score was included in the multivariate model, hyperglycemia, left anterior descending culprit artery, peak CK, and FP score remained independent predictors of high DE score (Table 3).

### Hyperglycemia and perfusion scores

Spearman rank correlation analysis showed a positive relationship between admission glycemia and perfusion scores (r = +0.34 and p < 0.001 for the FP score; r = +0.32 and p < 0.001 for the DE score). Categorization of patients according to the presence of hyperglycemia showed that FP (4 [0–8] vs 10 [5-16]; p < 0.001) and DE scores (11 [6-19] vs 18 [14-26]; p < 0.001) were higher in patients with hyperglycemia. In contrast, there was no difference between the 2 groups for LVEF (53 [43–59] vs 50 [43–56] %; p = 0.38), EDV (148 [125–170] vs 142 [117–173] ml; p = 0.23) and ESV (69 [55–84] vs 68 [51–95] ml; p = 0.85).

## Discussion

The major findings of the present study are that admission hyperglycemia, in the setting of an acute STEMI, is associated with the extent of MO as assessed by CMR. Hyperglycemia was also an independent predictor of myocardial infarct size.

### Hyperglycemia and microvascular obstruction

First-pass hypoenhancement is recognized as closely defining the extent of MO [24], and is strongly associated with worse outcome after STEMI [25-27]. MO is a major factor involved in the development of the angiographic "no-reflow" phenomenon, and limits the benefits of reperfusion [28]. MO may result from leukocyte entrapment in capillaries [29], and from distal microembolization of platelets which aggregate and adhere to capillary walls following plaque rupture [30].

In the present study, admission hyperglycemia was a predictive factor for the extent of MO, as assessed by the FP score. To the best of our knowledge, this is the first study to analyse the relationship between acute hyperglycemia and MO assessed by CMR, after successful reperfusion. Nevertheless, these findings are in agreement with those of Iwakura et al, who investigated the impact of admission hyperglycemia and the no-reflow phenomenon, assessed by intracoronary myocardial contrast echocardiography immediately after successful reperfusion [5]. They found that hyperglycemia was the strongest predictive factor for no-reflow.

The potential mechanisms involved in this relationship have yet to be elucidated. Even so, acute hyperglycemia is associated with increased platelet activation and fibrinolysis [31], as well as plugging of capillaries by leukocytes [32]. Acute hyperglycemia may also attenuate endothelium dependent vasodilatation, and reduce collateral blood flow to the area at risk [33]. Moreover, hyperglycemia may reduce the impact of ischemic preconditioning, through the attenuation of mitochondrial adenosine triphosphate-regulated K channel activation [34]. All of these mechanisms could strongly interact to favor the development of MO. However, in our study MO was mainly related to myocardial infarct size; which could partly explain the relation observed between hyperglycemia and MO.

### Hyperglycemia and myocardial infarct size

CMR, with Late Gadolinium Enhancement is widely used to define infarct size. Extra-cellular contrast-agents accumulate in the myocardium due to increased capillary permeability oedema, increased extra-cellular space and slower kinetics in infarcted regions [24,35]. One major finding of the present work was the close relationship between admission glycemia and the extent of myocardial infarction assessed by the DE score, independently of pre-existing glucose metabolism (HbA_1c_) or markers of myocardial infarct size (peak CK value). The impact of stress hyperglycemia on the extent of myocardial necrosis is not fully understood. Acute hyperglycemia may increase the inflammatory response during STEMI, and could thus influence microvascular permeability and oedema [36]. Moreover, in a recent study, Timmer et al found that, in patients with STEMI, admission hyperglycemia was an important predictor of reduced epicardial flow in the infarct-related vessel before reperfusion therapy [37]. They also suggest that acute rather than chronic hyperglycemia is more important in predicting TIMI flow in patients after PCI.

Finally, admission hyperglycemia may not only be the cause of more severe myocardial damage, but also its consequence. Large myocardial infarcts are more likely to cause excessive secretion of catecholamine, which affect fatty acids and glucose homeostasis [38]. However, stress hyperglycemia is an imperfect marker of cardiac damage, as many other markers in addition to stress hormones contribute to glucose metabolism [2].

### Study limitations

Follow-up data were not available in this study, which attenuates the impact of the study conclusion.

For evaluation of perfusion and Late Gadolinium enhancement, we did not use a real quantitative method but a semi-quantitative visual method to grade the extent of transmural myocardial damage [11,22]. However, the 17 segment model, which has gained wide acceptance in scintigraphic myocardial perfusion imaging, is both easy to obtain and reproducible. Moreoever in a previous study we showed an excellent correlation and concordance between visual grading and planimetric evaluation of myocardial enhancement [21].

The correlation between glycemia and perfusion scores, although significant, was weak. This relation was consolidated by the analysis of determinants of supramedian perfusion scores (Table 1). However, the relationship between admission glycemia and FP or DE parameter needs to be demonstrated in larger study population.

## Conclusion

Abnormal glucose metabolism is strongly associated with MO and the extent of myocardial infarct in successfully reperfused STEMI, as assessed by CMR. These results emphasize the interest of aggressive glucose control at the acute phase of STEMI. Moreover, CMR could play a major role in evaluating the impact of glucose control on myocardial perfusion.

## Abbreviations

CK: Creatine Kinase;

CMR: Cardiac Magnetic Resonance Imaging;

DE: Delayed Contrast-Enhanced;

EDV: Left Ventricular End Diastolic Volume;

ESV: Left Ventricular End Systolic Volume;

FP: First Pass;

LVEF: Left Ventricular Ejection Fraction;

MO: Microvascular Obstruction;

PCI: Percutaneous Coronary Intervention;

STEMI: ST-Segment Elevation Myocardial Infarction;

TIMI: Thrombolysis in Myocardial Infarction.

## Competing interests

The author(s) declare that they have no competing interests.

## Authors' contributions

Each author have contributed significantly to the submitted work. Analysis and interpretation of data have been performed by AC, MZ, IL and CT; drafting of the manuscript by AC, MZ, YC, FB, and JEW; AL, PMW and BV have revised the manuscript for important intellectual content. All authors read and approved the final manuscript.
